# Learning coronary artery calcium scoring in coronary CTA from non-contrast CT using unsupervised domain adaptation

**DOI:** 10.3389/fcvm.2022.981901

**Published:** 2022-09-12

**Authors:** Zhiwei Zhai, Sanne G. M. van Velzen, Nikolas Lessmann, Nils Planken, Tim Leiner, Ivana Išgum

**Affiliations:** ^1^Department of Biomedical Engineering and Physics, Amsterdam University Medical Center, Location University of Amsterdam, Amsterdam, Netherlands; ^2^Faculty of Science, Informatics Institute, University of Amsterdam, Amsterdam, Netherlands; ^3^Amsterdam Cardiovascular Sciences, Heart Failure and Arrhythmias, Amsterdam, Netherlands; ^4^Diagnostic Image Analysis Group, Radboud University Medical Center Nijmegen, Nijmegen, Netherlands; ^5^Department of Radiology and Nuclear Medicine, Amsterdam University Medical Center, Location University of Amsterdam, Amsterdam, Netherlands; ^6^Department of Radiology, Utrecht University Medical Center, University of Utrecht, Utrecht, Netherlands; ^7^Department of Radiology, Mayo Clinic, Rochester, MN, United States

**Keywords:** coronary artery calcium scoring, unsupervised domain adaptation, convolutional neural network (CNN), coronary CTA, adversarial learning

## Abstract

Deep learning methods have demonstrated the ability to perform accurate coronary artery calcium (CAC) scoring. However, these methods require large and representative training data hampering applicability to diverse CT scans showing the heart and the coronary arteries. Training methods that accurately score CAC in cross-domain settings remains challenging. To address this, we present an unsupervised domain adaptation method that learns to perform CAC scoring in coronary CT angiography (CCTA) from non-contrast CT (NCCT). To address the domain shift between NCCT (source) domain and CCTA (target) domain, feature distributions are aligned between two domains using adversarial learning. A CAC scoring convolutional neural network is divided into a feature generator that maps input images to features in the latent space and a classifier that estimates predictions from the extracted features. For adversarial learning, a discriminator is used to distinguish the features between source and target domains. Hence, the feature generator aims to extract features with aligned distributions to fool the discriminator. The network is trained with adversarial loss as the objective function and a classification loss on the source domain as a constraint for adversarial learning. In the experiments, three data sets were used. The network is trained with 1,687 labeled chest NCCT scans from the National Lung Screening Trial. Furthermore, 200 labeled cardiac NCCT scans and 200 unlabeled CCTA scans were used to train the generator and the discriminator for unsupervised domain adaptation. Finally, a data set containing 313 manually labeled CCTA scans was used for testing. Directly applying the CAC scoring network trained on NCCT to CCTA led to a sensitivity of 0.41 and an average false positive volume 140 mm^3^/scan. The proposed method improved the sensitivity to 0.80 and reduced average false positive volume of 20 mm^3^/scan. The results indicate that the unsupervised domain adaptation approach enables automatic CAC scoring in contrast enhanced CT while learning from a large and diverse set of CT scans without contrast. This may allow for better utilization of existing annotated data sets and extend the applicability of automatic CAC scoring to contrast-enhanced CT scans without the need for additional manual annotations. The code is publicly available at https://github.com/qurAI-amsterdam/CACscoringUsingDomainAdaptation.

## 1. Introduction

In recent years, deep neural networks have achieved impressive performance on various medical image analysis tasks (1, 2). This success is highly associated with the use of large amounts of representative annotated training data. However, the dependence on such data sets limits the applicability of already trained and well-performing networks to non-representative data sampled from a different distribution, such as images acquired at different sites, on different scanners, and by different acquisition protocols. Hence, generalizing deep neural networks trained on specific data to test data originating from a different domain remains a major challenge.

The domain shift, i.e., differences in data distributions and types of data between training and test domains, can be addressed by unsupervised domain adaptation methods that transfer a model that was trained on the source domain in a supervised manner to the target domain where no labels are available (3, 4). The common idea of unsupervised domain adaptation methods is to align features extracted by a network between two domains, aiming to generate similar feature distributions for both domains (4, 5). To achieve this, an adversarial learning strategy can be used. In this case, the generator network is optimized to extract features with similar distribution for the two domains while the discriminator network is trained to distinguish features from these domains (6).

Several works have investigated methods for unsupervised approaches to domain shift problem for segmentation of cardiac images (7–10). Dou et al. (8) proposed an unsupervised adversarial domain adaptation network to transfer cardiac segmentation network between MRI and CT. In this work the feature distributions of source and target domains were aligned at multiple scales. Chen et al. (7) extended the work of Dou et al. by aligning the domains in both image and feature perspectives. This method was evaluated with cardiac segmentation and abdominal multi-organ segmentation between MRI and CT. Wu et al. (10) presented an unsupervised domain adaptation framework to adapt cardiac segmentation between MRI and CT. In this method, a novel distance metric was proposed to calculate the misalignment of feature distributions in latent space and enable explicit domain adaptation.

In this work, we address detection and quantification of coronary artery calcium (CAC scoring) in contrast-enhanced coronary CT angiography (CCTA). Our aim is to exploit large sets of already annotated data in CT scans without contrast enhancement and extend the applicability of CAC scoring to CCTA. Current CAC scoring protocols are performed in a highly standardized manner without injection of iodinated contrast. Coronary artery calcifications are identified as high density areas of ≥ 130 Houndsfield Units (HU) in the coronary artery (11). Manual CAC scoring can be tedious and time-consuming, therefore, automated CAC scoring methods have been proposed (12, 13). Recent methods using deep learning have demonstrated accurate performance (14, 15). Given that CAC scoring is commonly performed in non-contrast CT (NCCT), automated methods have mostly focused on application in these scans. While earlier methods focused on a single type of NCCT scans (16–18) recent studies showed that the methods can generalize to diverse types of NCCT data. In a large-scale study containing data of 7,240 subjects, Van Velzen et al. (19) trained and evaluated a method proposed by Lessmann et al. (16) with different types of NCCT scans including scans from different hospitals, multiple scanners and multiple image acquisition protocols and demonstrated a good agreement between automated and manual scoring. Subsequently, Zeleznik et al. (20) demonstrated the robustness of a deep learning system for automated CAC scoring on routine cardiac gated and non-gated NCCT of 20,084 individuals.

In addition to CAC scoring in NCCT, CAC can be quantified in CCTA (21) and consequently, a number of methods automating the process have been developed (22–25). In a clinical cardiac CT exam, commonly cardiac NCCT is acquired first to determine the calcium score, which is followed by the acquisition of CCTA to detect presence of non-calcified plaque and stenosis in the coronary arteries. However, the amount of calcified plaque extracted from CCTA scans allows accurate cardiovascular risk stratification (22, 24). Hence, when the scan without contrast is not available, calcium scoring in CCTA may allow determination of patient's cardiovascular risk and thus allow better utilization of the already acquired data. Furthermore, performing CAC scoring in CCTA could allow omitting acquisition of the NCCT and thereby reduce the radiation dose to the patient and save scan time (24, 25). Coronary artery calcium scoring in CCTA differs substantially from scoring in NCCT as the contrast material enhancing the coronary artery lumen typically exceeds the threshold (130 HU) used for CAC scoring in NCCT. Therefore, automatic methods trained on NCCT are not directly applicable to CCTA scans. Training the deep learning method with extra annotated CCTA data may improve its applicability to CCTA. However, manually annotating a large amount of representative training data is tedious and time consuming. To address this, in this study, we investigate the feasibility of adapting a CAC scoring network trained on a large set of labeled NCCT scans (16, 19) to unlabeled CCTA scans using unsupervised domain adaptation. For this, we investigate a cross-domain approach described by Dou et al. (8) to enable CAC scoring in CCTA without annotations while utilizing NCCT with available manual annotations.

## 2. Materials

### 2.1. Image data

This study includes three data sets. First, a data set of *labeled* low-dose chest NCCT scans from the National Lung Screening Trail (NLST) was used. The NLST enrolled 53,454 current or former heavy smokers aged 55–74 in the United States (26). In our previous study, a set of 1,687 baseline chest NCCT scans was selected (16). This set was designed to be diverse with respect to scanner model and reconstruction algorithm. The selected scans were acquired on 13 different scanner models in 31 hospitals. These chest NCCT scans were acquired with breath hold after inspiration and using a tube voltage 120 or 140 kVp, depending on the subjects weight. Scans were reconstructed to 0.49–0.98 mm in-plane resolution, 1–3 mm slice thickness, and 0.6–3 mm increment. For our work, all scans were resampled to 3 mm slice thickness and 1.5 mm increment, following earlier studies (16).

Second, a mixed set of *labeled* cardiac NCCT and *unlabeled* CCTA scans was used. Specifically, 200 labeled cardiac NCCT scans were acquired in clinical patient workup at University Medical Center Utrecht, The Netherlands (19, 27) and 200 unlabeled CCTA scans were acquired at Amsterdam University Medical Center location University of Amsterdam, The Netherlands. The cardiac NCCT scans were acquired with a Philips Brilliance iCT 256 scanner, with ECG synchronization and 120 kVp tube voltage. Scans were reconstructed to 0.29–0.49 mm in-plane resolution, 3 mm slice thickness, and 1.5 increment. The CCTA scans were acquired with a Siemens Somatom Force CT Scanner, with ECG synchronization and 70–120 kVp tube voltage. Scans were reconstructed to 0.22–0.46 mm in-plane resolution, 0.6 mm slice thickness, and 0.4 mm increment.

Third, a data set of *labeled* 313 CCTA scans from Amsterdam University Medical Center location University of Amsterdam, The Netherlands was used to evaluate the CAC detection on the target domain (CCTA test set). These CCTA scans were acquired with the Siemens Somatom Force CT Scanner, with ECG synchronization and 70–120 kVp tube voltage. Scans were reconstructed to 0.19–0.77 mm in-plane resolution, 0.6–1 mm slice thickness, and 0.4 mm increment.

### 2.2. Manual reference annotations

Manual reference labels of CAC were available from previous studies for the low-dose chest NCCT scans in the NLST data set(16) and the cardiac NCCT in the mixed set (19). The labeling was performed semi-automatically: all regions of ≥ 3 adjacent voxels with a CT value above 130 HU were shown as overlay. An observer manually identified lesions and labeled them according to their anatomical location, i.e., left anterior descending artery (LAD), left circumflex artery (LCX), or right coronary artery (RCA) (19). Given that chest CT without ECG synchronization does not allow visualization of the left main (LM) artery, CAC in the LM was labeled as LAD. Examples of chest NCCT slices and manual reference annotations are shown in the Supplementary Figure S1.

For the 200 CCTA scans in the mixed set, reference labels of CAC were not available. Hence, for the CCTA scans from the CCTA test set, CAC was manually annotated with a semi-automated method as either LAD, LCX, or RCA. This was done using an in-house developed software designed in MevisLab 3.2 (28). In agreement with manual labeling in NCCT, CAC in the LM was labeled as LAD. Because the standard 130 HU threshold for CAC detection in NCCT can not be used in CCTA, we used scan specific thresholds, following earlier studies (25, 29). For this, a region of interest (ROI) defined by a bounding box with a size around 35 × 36 × 44 voxels in the ascending aorta at the level of the origin of the left coronary artery was manually selected. Subsequently, the mean *mean*_*ROI*_ and standard deviation *STD*_*ROI*_ from the CT values of the voxels within the ROI were used to compute a scan specific threshold *mean*_ROI_+3*STD*_ROI_. Using this threshold, each coronary artery calcification was manually identified by a mouse click on the lesion. Subsequently, all connected voxels in the lesion above the scan specific threshold were marked as CAC in LAD, LCX, or RCA using 3D connected component labeling considering six-voxel connectivity. Examples of CCTA slices and manual reference annotations are shown in the Supplementary Figure S2.

In this study, NCCT scans (both chest and cardiac) are considered the source domain and CCTA scans are representing the target domain. The NCCT scans with CAC annotations from the NLST data set were used to train the CAC detection network on the source domain. The mixed set of labeled cardiac NCCT (source domain) and unlabeled CCTA (target domain) was used to train our unsupervised domain adaptation method. The labeled CCTA scans (target domain) in the CCTA test set were only used to evaluate the CAC detection on the target domain. The description of data sets and their usage are illustrated in Table 1.

**Table 1:** Description of data and corresponding usage.

**Scan type**	**#Scans**	**Reference**	**Domain**	**Usage**
Chest NCCT	1,687	✓	Source	Training CAC scoring on source domain
Cardiac NCCT	200	✓	Source	Training unsupervised domain adaptation
CCTA	200	✗	Target
CCTA	313	✓	Target	Testing CAC scoring on target domain

## 3. Methods

**Figure 1:**
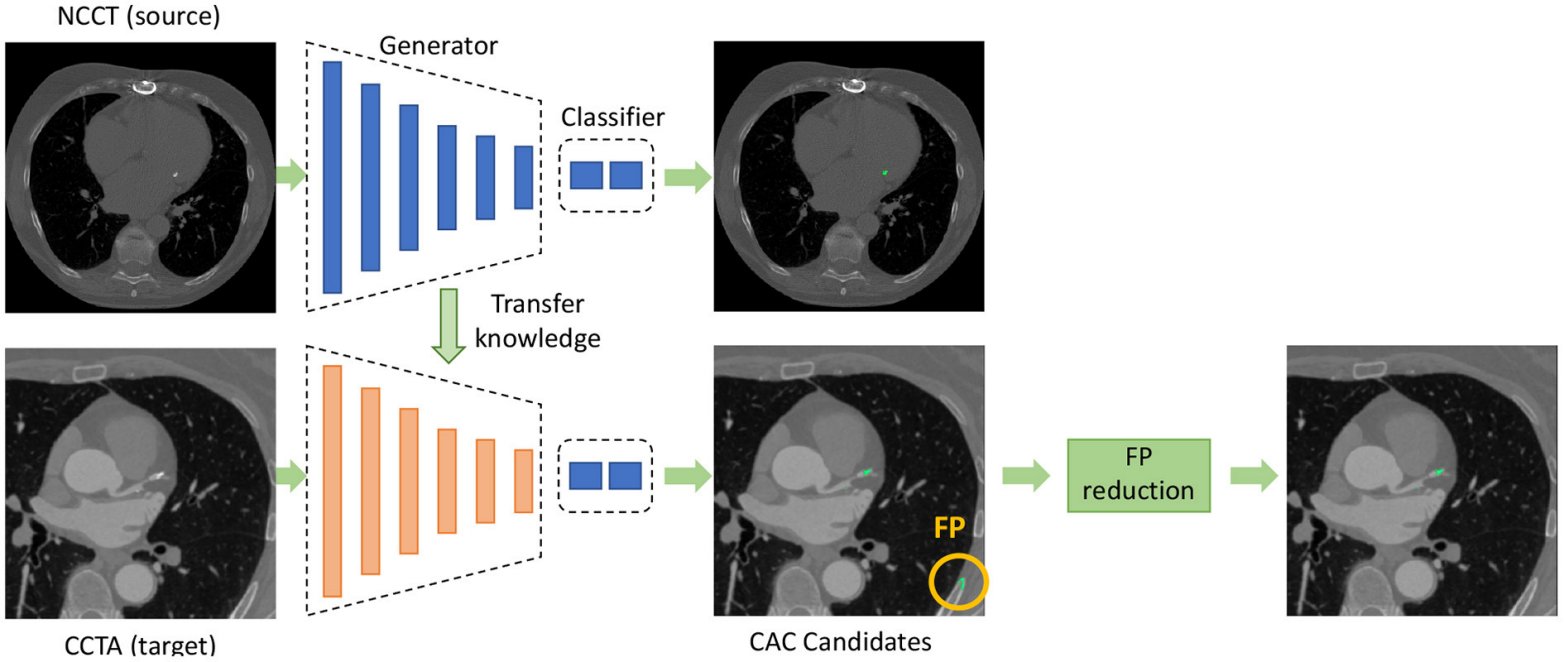
Overview of the proposed method for coronary artery calcium (CAC) detection in CCTA. The CNN for CAC detection is divided into a feature generator and a classifier. The feature generator is trained on source domain and is adapted to the target domain using unsupervised domain adaptation. The classifier in the target domain is reused from the source domain. After detection of CAC candidates using the CNN, false positive (FP) reduction is applied to remove FP detections.

A CNN is used for detecting CAC candidates in CCTA scans that is followed by false positive (FP) reduction, as shown in Figure 1. The CNN, which is trained on labeled NCCT data is adapted for application in CCTA using unsupervised domain adaptation. False positive reduction is performed by limiting the detected lesions to plausible CAC location and size.

### 3.1. CAC detection in CCTA with unsupervised domain adaptation

Unsupervised domain adaptation aims to transfer a model trained with data from a source domain with labels $D_{s}={\left(X_{s}^{i},Y_{s}^{i}\right)}_{i=1..n_{s}}$ to a target domain without labels $D_{t}={\left(X_{t}^{i}\right)}_{i=1..n_{t}}$, where D represents domain, *X* represents images and *Y* represents labels. As proposed by Dou et al. (8), we use an adversarial training strategy to adapt the CNN to the target domain. In our application, a large set of chest NCCT scans with CAC labels is available, and hence, we aim to transfer the knowledge from NCCT to CCTA for CAC scoring. Therefore, the CAC scoring CNN trained with *labeled* low-dose chest NCCT scans is transfered to CCTA using adversarial domain adaptation.

We used our previous CAC scoring method described by Lessmann et al. (16) that has been trained and evaluated with a large set of low-dose chest NCCT data. The method consists of two sequential convolutional neural networks (CNN). The first CAC scoring CNN detects CAC candidates and labels them according to their anatomical location, i.e., as CAC in LAD, LCX, or RCA. The second CNN reduces the number of false positive detections. In our current work, only the first CNN is used to transfer knowledge obtained by training the network with NCCT to enable application in CCTA data using unsupervised domain adaptation.

To adapt the CAC detection network(16) from the source domain to the unlabeled target domain, we aim to align the distributions of extracted features from the two domains following the work by Dou et al. (8). For this, we divide the CAC detection network into two parts: a feature generator *G*(·) and a classifier *C*(·), as shown in Figure 1. The *G*(·) maps input images into feature representations in the latent space and the *C*(·) predicts the output class from the feature representations. The early layers of the network which are used for feature extraction are mostly related to the domain, while the deeper layers are mostly task-specific and learn semantic-level features for conducting the predictions (8, 30). Hence, we adapt the feature generator *G*(·) trained with NCCT to enable application in CCTA with adversarial domain adaptation, and we reuse the classifier *C*(·) as originally trained.

**Figure 2:**
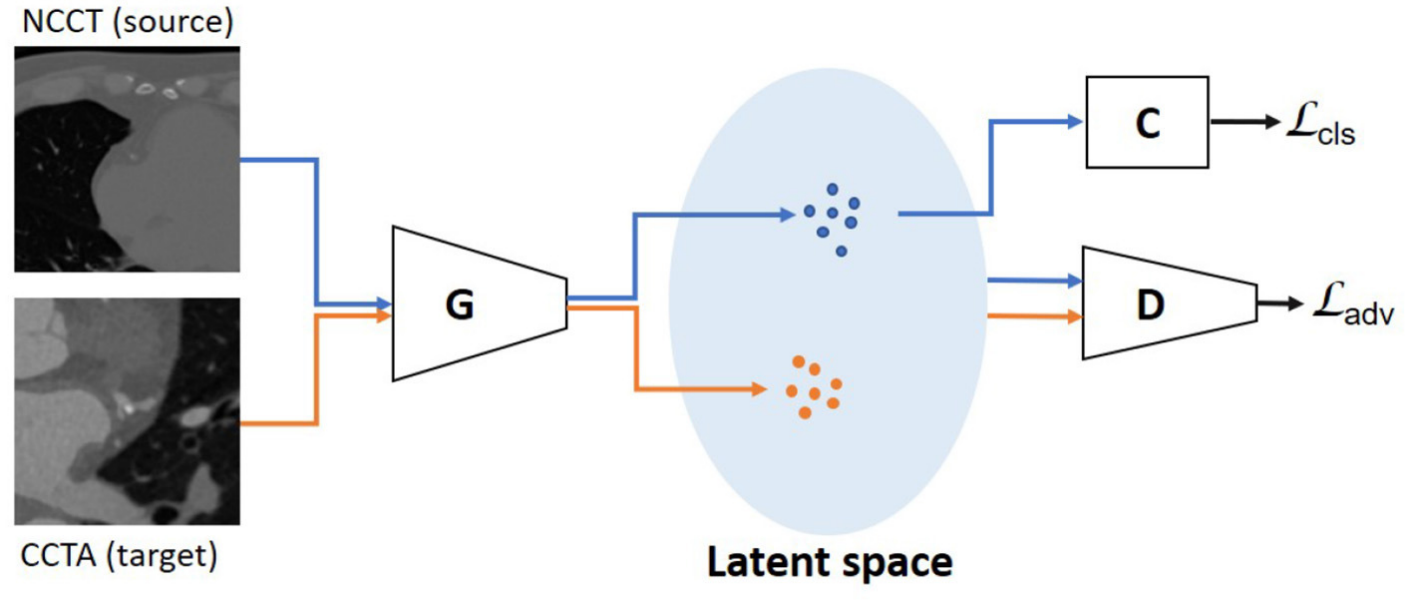
Unsupervised domain adaptation with *unpaired* data is performed using an adversarial learning strategy. The discriminator is optimized to distinguish the features from NCCT (source) domain and CCTA (target) domain. The generator is trained to extract features with similar distributions for the two domains. The blue dots in latent space represent features from the source domain, the orange ones from the target domain. The $L_{adv}$ is used as the objective function and the $L_{cls}$ is used as a constraint, which is determined on the source domain using the classifier.

To enable adversarial learning, we design a discriminator *D*(·) to identify whether the features are from the source domain or the target domain. While the feature generator *G*(·) aims to extract features with similar distributions for both domains, the *D*(·) discriminates between the two domains (Figure 2). The adversarial loss based on the differences in feature distribution between the two domains is formulated as:


(1) $$\begin{array}{r} L_{adv}=E_{x_{t}\in D_{t}}log\left(D\left(G\left(x_{t}\right)\right)\right)-E_{x_{s}\in D_{s}}log\left(D\left(G\left(x_{s}\right)\right)\right) \end{array}$$

where *G*(·) is optimized to minimize the adversarial loss, and *D*(·) is optimized to maximize the same loss. The generator *G*(·) is optimized based on the objective function calculated from the discriminator *D*(·), which can lead to an incorrect optimization forgetting the classification task. That means the features extracted by the trained *G*(·) can fool the *D*(·). However, these features are not beneficial for the final classification task *C*(*G*(·)). For cross domain learning with *paired* data, the alignment loss in feature space, such as *L*1(*G*(*x*_*s*_), *G*(*x*_*t*_)) or *L*2(*G*(*x*_*s*_), *G*(*x*_*t*_)), can be used as a constraint for the generator optimization (31). For cross-domain learning with *unpaired* training data as in our case, such an alignment loss in feature space can not be used as a constraint for the generator optimization. In this work, the images were not registered to a common space either. Instead, as proposed in the work by Chen et al. (4), we use a classification loss in the source domain $D_{s}$ as constraint to stabilize the training and avoid catastrophic forgetting.

The classification loss is formulated as:


(2) $$\begin{array}{r} L_{cls}=L_{CE}\left(C\left(G\left(X_{s}\right)\right),Y_{s}\right) \end{array}$$

where *L*_*CE*_ is the cross-entropy loss, *X*_*s*_ and *Y*_*s*_ are the images and the corresponding reference labels on the source domain. During training, the *D*(·) is trained to maximize the objective of $L_{adv}$, while the *G*(·) is optimized to minimize the objective of $L_{adv}$ and $L_{cls}$. These are formulated as:


(3) $$\begin{array}{l} {}_{\;D}^{\max{ℒ}_{adv}}\\ {}_{\;G}^{\min{ℒ}_{adv}+\alpha {ℒ}_{cls}} \end{array}$$

where α is a hyper-parameter for balancing the two loss terms. It is set to 2.0 in this work, based on a grid search strategy.

### 3.2. FP reduction

To identify CAC lesions, 3D connected component labeling is performed from the detected voxels and the scan specific threshold (25, 29). To remove potential false positive detections, detected lesions smaller than 1 mm^3^ are discarded as those are likely noise voxels. Similarly, detected lesions larger than 500 mm^3^ are discarded as those exceed the expected CAC volume (27). In addition, lesions detected outside the heart are discarded. For this, the heart volume is defined by segmentation of cardiac chambers, as described by Bruns et al. (32) which was trained with CCTA scans of 12 patients scanned for transcatheter aortic valve implantation (SOMATOM Force, Siemens, 70–120 kVp, 310–628 mAs, in-plane resolution 0.31–0.61 mm, slice thickess 0.31–0.61 mm, slice increment 0.45 mm). No additional changes or fine tuning for the data in this current study was performed. Subsequently, the segmentation of cardiac chambers was dilated by a sphere as a structuring element with diameter of 10.0 mm to ensure the heart wall and coronary arteries are included in the segmentation.

### 3.3. Evaluation

To evaluate the performance of CAC scoring on CCTA, the volume-wise and lesion-wise performance was determined by comparing automatically detected CAC with the manually annotated reference. Since the typically used Agatston score (11) is not applicable for CAC quantification in CCTA, the volume score was used. The evaluation was performed for total CAC and separately for CAC in LAD, LCX, and RCA. Both the volume-wise and lesion-wise performance was evaluated using sensitivity, false-positive (FP) rate, and F1 score (16). The agreement of calcium volume and number of lesions between the automatic detection and the reference labels was determined with Spearman correlation coefficients. Finally, the agreement between automatic volume scores and manual reference volume scores was assessed by examining Bland-Altman plots including 95% limits of agreement. Since errors tend to increase with increasing CAC volume, the variation of absolute differences between automatic and manual scores was modeled using regression for nonuniform differences(33). Because the absolute differences have a half-normal distribution, the modeled absolute differences were multiplied by 1.96 × (π/2)^0.5^ to obtain the 95% limits of agreement.

## 4. Experiments and results

### 4.1. CAC scoring on CCTA

First, we retrained the two-stage CNNs for CAC detection (16) with the *labeled* chest NCCT data as the source domain. For this, the 1,687 NCCT scans in the NLST data set were randomly divided into 60% training set (1,012 scans), 10% validation set (169 scans), and 30% test set (506 scans). As originally reported (16), during the training, categorical cross-entropy was used as loss function, Adam was used as optimizer with a learning rate of 5 × 10^−4^. The first CNN was trained with three orthogonal (axial, sagittal and coronal) patches of 155×155 pixels and the second CNN with three orthogonal patches of 65×65 pixels (16). Randomized patch extraction was used as augmentation for training.

Next, to stabilize adversarial training in the unsupervised domain adaptation, the generator was initialized with the weights of the CAC scoring model trained with the chest NCCT data from the NLST dataset. The unsupervised domain adaptation method was trained with the mixed dataset of *labeled* cardiac NCCT data from source domain and *unlabeled* CCTA data from target domain. When performing unsupervised domain adaptation with mixed data containing *labeled* cardiac NCCT and *unlabeled* CCTA scans the method achieved sensitivity of 0.78 in CCTA (Table 2). For comparison, the sensitivity of 0.53 was achieved when unsupervised domain adaptation was performed with mixed data containing *labeled* chest NCCT and *unlabeled* CCTA scans. *Labeled* cardiac NCCT data was chosen because these scans resemble CCTA scans more than chest NCCT. *Unlabeled* CCTA were used as *unlabeled* data from the target domain. To obtain a reliable discriminator, the discriminator was solely pretrained for 1,000 iterations first. Thereafter, the generator and discriminator were optimized together by training alternately. Specifically, the generator was optimized one iteration after every 20 iterations of the discriminator, according to the heuristic rules of training a Wasserstein GAN (34). Following the standard for adversarial training (34, 35), the discriminator was kept in a compact space. To enforce this constraint, the weights were clipped between [−0.1, 0.1]. The RMSProp optimizer was used to optimize the discriminator with a learning rate of 5 × 10^−4^, and the generator with a learning rate of 5 × 10^−5^, respectively (36). The optimal hyperparameters were determined by grid search. The adversarial learning was trained for 200 epoch. The networks were implemented in PyTorch (37). All the training was trained on NVIDIA GeForce RTX 2080 Ti.

**Table 2:** Results of the automatic CAC scoring evaluated by volume-wise sensitivity, FP volume per scan, and F1-score between automatic detection and manual reference.

		**NCCT [506]**	**CCTA [313]**			
	$L_{adv}$	✗	✓	✓	✓	✗
	$L_{cls}$	✗	✓	✓	✗	✗
	FP reduction	✗	✓	✗	✗	✗
CAC	Sensitivity	0.89 (0.25)	0.80 (0.32)	0.78 (0.33)	0.68 (0.38)	0.41 (0.48)
	FP volume/scan	73.6 (141)	19.8 (60.6)	64.5 (150)	25.8 (70)	132 (205)
	F1	0.66 (0.37)	0.66 0.38	0.41 (0.40)	0.49 (0.41)	0.16 (0.36)
LAD	Sensitivity	0.92 (0.21)	0.89 (0.27)	0.86 (0.28)	0.79 (0.33)	0.47 (0.48)
	FP volume/scan	31.6 (79.6)	13.9 (45.5)	44.5 (118)	20.2 (54.4)	55.8 (90.5)
	F1	0.79 (0.34)	0.74 (0.37)	0.48 (0.42)	0.56 (0.42)	0.24 0.41
LCX	Sensitivity	0.88 (0.29)	0.74 (0.44)	0.71 (0.45)	0.71 (0.46)	0.66 (0.48)
	FP volume/scan	19.7 (55.6)	0.13 (1.13)	0.17 (1.01)	0.02 (0.31)	1.60 (0.30)
	F1	0.67 (0.42)	0.74 (0.44)	0.69 (0.46)	0.70 (0.46)	0.66 (0.48)
RCA	Sensitivity	0.89 (0.26)	0.87 (0.30)	0.87 (0.31)	0.80 (0.38)	0.67 (0.47)
	FP volume/scan	30.1 (73.4)	6.80 (35.6)	21.3 (78.1)	6.64 (35.6)	77.6 (157)
	F1	0.65 (0.42)	0.73 (0.41)	0.52 (0.46)	0.68 (0.44)	0.31 (0.46)

The method with different settings (using adversarial loss and classification loss in the CAC detection network, and false positive reduction stage) is tested on chest NCCT data and CCTA data. FP volume/scan is given in mm^3^.

The results are shown as average (standard deviation) for total CAC as well as for LAD, LCX, and RCA separately. $L_{adv}$, adversarial loss; $L_{cls}$, classification loss; CAC, coronary artery calcification; LAD, left anterior descending artery; LCX, left circumflex artery; RCA, right coronary artery.

**Figure 3:**
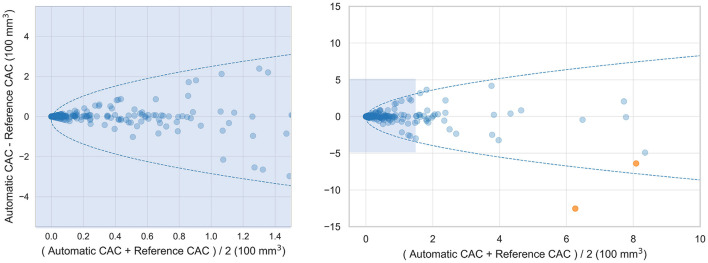
Bland-Altman plots comparing automatically detected CAC volume with the manual reference volume. 95% limits of agreement are represented by the formula: *Difference* = ±1.96 × (π/2)^0.5^×(*b*+*a*×*Mean*^0.5^), with *a* = 10.9 and *b* = −17.8. Two outlier cases are colored orange. The Bland-Altman plot of lesions with volume less than 150 mm^3^ is shown on the left and all lesions is shown on the right.

To establish the performance of the CNN adapted from NCCT to CCTA, the network was evaluated with the 313 labeled CCTA test scans. The adapted CNN obtained an average volume-wise sensitivity of 0.78, an average FP volume per scan of 73.9 mm^3^ and an F1-score of 0.41. After the FP reduction, the proposed method achieved an average volume-wise sensitivity of 0.80 with an average FP volume per scan of 19.8 mm^3^, and F1 of 0.66. There were 36 patients without CAC but with FP detected by the proposed method, with an average FP volume per scan of 40 mm^3^. The Spearman correlation between automatically detected and reference CAC volume was 0.73. The Bland-Altman plots comparing automatically detected CAC volume with manually annotated reference are illustrated in Figure 3.

**Figure 4:**
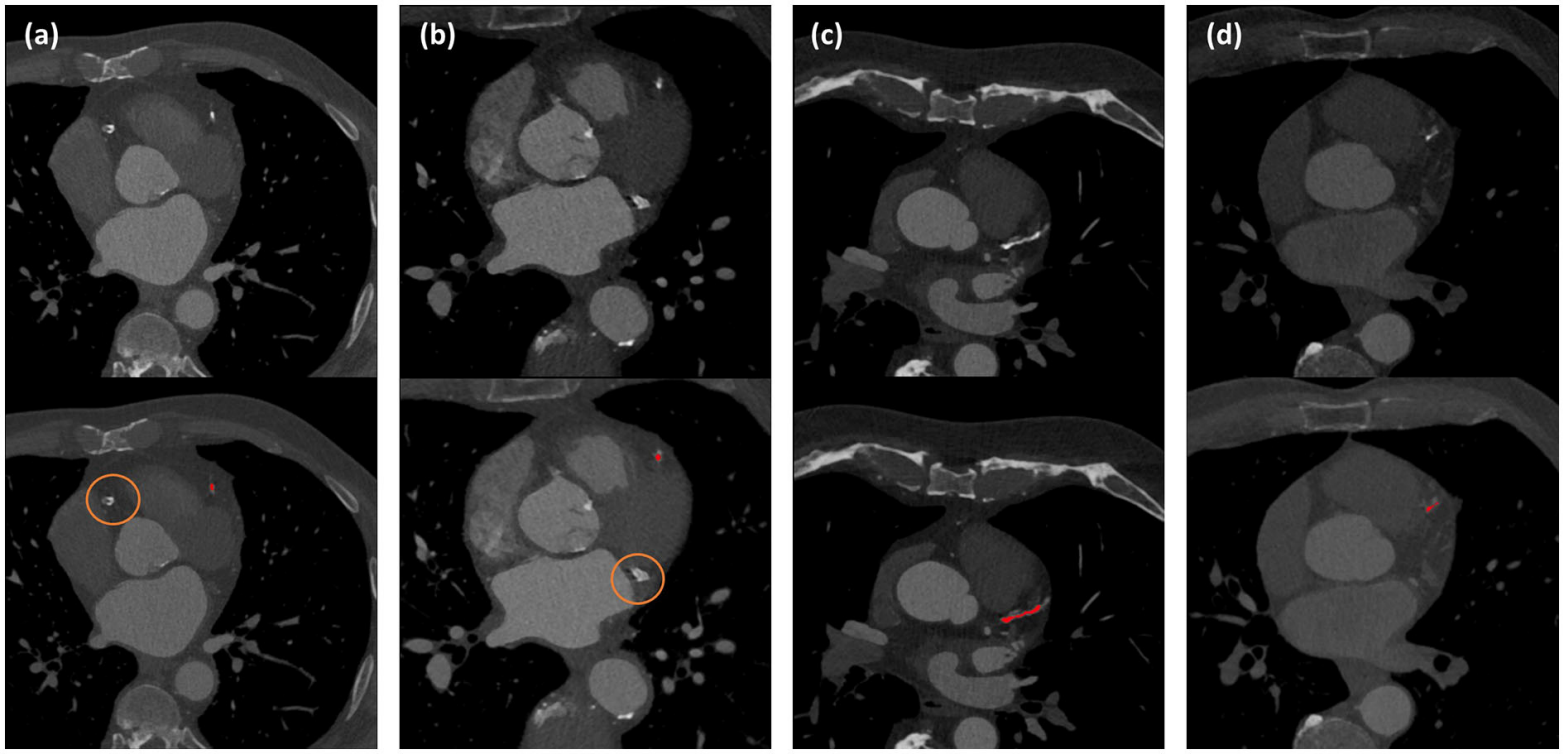
Automated CAC detection results in CCTA scans of four patients. The images in the first row show CCTA slices and the detected CACs are shown as overlay in the second row. Panels **(a)** and **(b)** illustrate the two largest outliers shown by orange dots in Figure 3, and false negative CAC are indicated by orange circles. Panels **(c)** and **(d)** show two cases with correct automatic CAC detections.

Coronary CT angiography slices and corresponding automatic CAC detections for two outliers cases (marked orange in Figure 3) are shown in Figures 4a,b. In addition, two representative cases from the labeled CCTA test set are shown in Figures 4c,d. For lesion-wise evaluation, the proposed method achieved an average sensitivity of 0.79 and FP lesion per scan of 1.06. The correlation between the number of automatically detected and manually annotated reference lesions was 0.69.

### 4.2. Ablation study

To establish whether our retraining of the original CAC scoring network on the source domain led to adequate performance, the CAC scoring network was evaluated on NLST test set (Section CAC scoring on CCTA) and compared with the originally reported results (16). Results are listed in Table 2 (column 3 showing NCCT results). Our retained network obtained a sensitivity of 0.89, an average FP volume of 73.6 mm^3^ per scan and F1 of 0.66. The sensitivity is in agreement with the results (0.84 - 0.91) reported in the original work (16), while the originally reported FP rate (40.7–62.8 mm^3^) and therefore F1 (0.84–0.89) slightly outperform our results.

To evaluate the performance of the two-stage CAC scoring networks trained on NCCT to CCTA, the trained CNNs was directly applied to CCTA test scans without adversarial domain adaptation learning. This led to an average sensitivity of 0.41, an average FP volume per scan of 139.7 mm^3^, and F1 of 0.16 (Table 2, column 7 showing the CCTA results). Subsequently, adding FP reduction led to an average sensitivity of 0.43, an average FP volume of 0.58 mm^3^ and F1 of 0.41. Note that FP reduction stage slightly improved the sensitivity as the region-growing algorithm (38) used to define the lesions from the voxels detected by the CNN may improve lesion segmentation and lead to better agreement with manual reference that used the region-growing algorithm to define CAC lesions.

To investigate the benefit of using the adversarial loss and classification loss for domain adaptation, and FP reduction, additional experiments were performed. The proposed method obtained a volume-wise sensitivity of 0.80, average FP volume per scan of 19.8 mm^3^, and F1 of 0.66. Without FP reduction, the volume-wise sensitivity decreased to 0.78, average FP volume per scan increased to 64.5 mm^3^ and consequently, F1 score decreased to 0.41. Furthermore, removing the classification loss $L_{cls}$ from the objective function resulted in the volume-wise sensitivity of 0.68, average FP volume per scan of 25.8 mm^3^, and F1 of 0.49. Finally, as described above, removing the adversarial loss $L_{adv}$ (i.e., without adversarial domain adaptation learning) led to sensitivity of 0.41, FP volume of 139.7 mm^3^ per scan, and F1 of 0.16. Detailed results are listed in Table 2 columns 4–7.

### 4.3. Comparison with previous work

The performance of the proposed method was compared with previously published methods that use deep learning for CAC scoring in CCTA scans (22–25). Wolterink et al. (25) proposed a method that employed paired CNNs for CAC scoring. The first CNN was used to identify CAC-like voxels and the second CNN was used to reduce CAC-like negatives. Fischer et al. (22) proposed a method that firstly detected the coronary artery centerlines and then identified CAC in cross-sectional images along the detected centerlines using long short-term memory (LSTM). In the study by Liu et al. (23), a vessel focused 3D CNN was proposed for CAC detection. The coronary arteries were firstly extracted and straightened volumes were reformed along the coronary arteries. Thereafter, a CNN was used for CAC detection. The results as reported in the original work are listed in Table 3. These demonstrate that our unsupervised method achieved competitive performance. Given that the original implementations of these earlier studies are not publicly available, the compared methods the results should be used as indication only.

**Table 3:** Comparison with previously published results on automated coronary artery calcium scoring on CCTA.

			**Lesion-wise evaluation**			**Volume-wise evaluation**		
**Method**	**# train**	**# test**	**Sensitivity**	**FP lesion**	**F1**	**Sensitivity**	**FP volume**	**F1**
Wolterink et al. (25)	150	100	0.71	0.48	–	–	–	–
Liu et al. (23)	80	20	–	–	–	0.85	–	0.83
Fischer et al. (22)	232	194	0.92	0.20	–	–	–	–
Ours	–	313	0.79	1.06	0.66	0.80	19.8	0.66

The number of labeled CCTA scans used for training (# train) and testing (# test) are listed. Performance [sensitivity, false positives (FP) per scan and F1-score] using CAC lesions and volume are given.

## 5. Discussion

In this work, we have utilized an unsupervised domain adaptation method described by Dou et al. (8) employing a CNN architecture which enables CAC scoring in CCTA while learning from annotated non-representative CT scans without contrast and representative CCTA without reference annotations. For this, the first-stage CNN as previously designed by Lessmann et al. for CAC scoring (16) is divided into a feature generator and a classifier. The feature generator is adapted from NCCT to CCTA through adversarial unsupervised domain adaptation and the classifier trained on NCCT is reused. An adversarial loss and classification loss on source domain are used as the objective function. The results demonstrate that the method achieves a competitive performance.

Like previous methods for automatic calcium scoring, our method consists of two distinct stages. In the first stage, a CNN for CAC detection and labeling in non-contrast chest CT from previous work (16) is adjusted for the CAC scoring in CCTA. The ablation study showed that our retraining of the CAC detection CNN did not lead to the same performance reported in the original manuscript (16). However, there are several differences. First, although training and test scans originate from the same set, exact division on the scans into training and test set differs. Second, the original work reported results separately for sharp and soft kernel CT reconstructions, while we did not distinguish between these. Like in the original work, a second stage is used to reduce the number of false positives. Using the described approach for CAC scoring in CCTA, simple image processing (restricting allowed volume of CAC, limiting the analysis to the volume of interest) substantially reduced false positive detections. Nevertheless, retrospective analysis showed that occasionally false positives remain inside heart and in the coronary arteries with high HU value. Visual analysis of the results showed small false positive detections in the distal RCA representing contrast material. This is also reflected in the limited Spearman correlation coefficient between the detected and reference lesions. This might be due to the varying contrast levels of CCTA, where parts of the coronary artery lumen had a very high HU value. Likely, locally defined threshold for the extraction of CAC would alleviate the problem. Future research should investigate whether this would would benefit the overall performance. In few cases false positive detections were representing extra-coronary calcifications. Those were aortic calcification in the vicinity of the coronary ostia or calcifications in the aortic valves, which is not uncommon to automatic calcium scoring methods(19).

Retrospective analysis of the outliers shown in Figures 3 and 4 showed that in one case, a large CAC in the RCA (625 mm^3^) was detected by the CNN but removed in the FP reduction stage because its volume exceeded the maximum expected CAC volume. In the other case, large CAC in LCX (313 mm^3^) was not detected by the CNN. In our training set, median (Q1, Q3) CAC was 7.1 (1.6, 29.2) mm^3^ and 95th percentile was 188 mm^3^. This shows that the volumes of our false negatives substantially exceeded CAC examples in the training set. Adding examples of large CAC lesions in the training set or learning specifically focused on rare CAC examples might improve the performance.

To train the CNN for detection and labeling of CAC, three different data sets were used. First, we reused the CNN trained on a large set of labeled chest CTs without contrast enhancement. To achieve unsupervised domain adaptation, non-representative labeled cardiac CT without contrast and representative unlabeled CCTA were used. Future work could investigate the optimal size of each set and the optimal way of injecting different data into the training, e.g., training the CNN with different non-contrast CT scan types, refinement with specific data or introducing different data in the domain adaptation stage.

To make the cross domain training stable with unpaired data, the classification loss on the source domain was used. For cross domain learning with paired data, a feature-wise loss could be used (31). Given that we don't have paired data or register the images to a common space, this kind of loss is not applicable in our study. In our work, the feature generator was adapted from source domain to target domain, however, the classifier was directly reused. This could be done even though the input images to feature generator are from different domains because the classifier performs the same task with aligned feature distributions.

To transfer the knowledge of CAC detection from NCCT to CCTA, unsupervised domain adaptation was used. When a limited set of annotated training data from the target domain is available, it is common to pretrain the network with labeled data from the source domain and fine-tune the network with this small set (30, 39). In our case, annotated training data from the target domain is not available and unsupervised domain adaptation allows the training with labeled data from the source domain and unlabelled data from the target domain. Future work could investigate whether a small set of annotated images from the target domain may benefit the performance, possibly also by combining transfer learning approaches with unsupervised domain adaptation.

In this study, following the work by Dou et al. (8), the knowledge about CAC detection was transferred from NCCT to CCTA by aligning the feature distributions between the two domains. However, Chen et al. (7) performed unsupervised domain adaptation by aligning the domains in both image and feature perspectives. The image alignment was used to transform the image appearance and narrow the domain shift between source and target domains. However, we opted for feature alignment only because lack of visible anatomical boundaries in non-contrast scans (arteries, cardiac chambers) to guide the image registration renders image alignment a highly challenging task. Moreover, very small CAC may disappear due to registration, which would not be beneficial for learning.

Comparing the proposed method with previously published deep learning methods on CAC scoring in CCTA scans showed that the proposed method achieved a competitive sensitivity. However, the number of false positive detections did not reach the performance of supervised methods. Methods (22, 23) that limited the ROI for CAC scoring with coronary artery extraction, achieved a lower number of FP detections. Future research could investigate whether limiting the the analysis to the vicinity of the coronary arteries like proposed by Fischer et al. (22) and Liu et al. (23) would be beneficial. For this, tracking the coronary artery centerline (40) could be used.

Bland-Altman plot shown in Figure 3 shows heteroskedastic-like behavior of CAC scores. This behavior is not uncommon for CAC scoring methods, because typically errors tend to increase with higher CAC scores (19, 24). False negative detections tend to be larger in patients with higher calcium burden, possibly because their lesions tend to be larger. Moreover, larger false positive detections often consist of non-coronary calcifications, e.g., aortic calcifications in the vicinity of the coronary ostia or cardiac valves, which are also typically larger in patients with a higher coronary calcium burden. To calculate the 95% confidence intervals of the Bland-Altman plots we accounted for the heteroskedastic behavior by modeling the variation in absolute differences (33).

While CCTA scans are mainly made to provide important information on the presence and the amount of non-calcified plaque and stenosis, cardiac CT scans without contrast enhancement are the reference modality for quantification of calcified coronary artery plaque. Hence, limitation of our method is its ability to quantify calcified plaque in CCTA only. To fully exploit information contained in CCTA, our further work will focus on extending the method to quantification of calcified and non-calcified plaque and stenosis.

In this work, the unsupervised domain adaptation method was trained with 200 NCCT scans and 200 CCTA scans. Like with any machine learning methods, training the unsupervised domain adaptation method with more scans that include more diversity would likely lead to more accurate performance. Finding the optimal set size should be a topic of future research.

In the literature, a wide range in inter-observer agreement for CAC quantification in CCTA has been reported. Specifically, 11% variability in CAC volume when utilizing a scan-specific threshold (41) and 13–25% when using manual delineation of CAC (42). Moreover, correlation of CAC volume between observers of 0.89–0.98 has been reported (42, 43). In the current study the variability between automatic and reference scores was 21%, with a correlation of 0.73. Given that no clinically used risk categories are defined based on CAC volume or other CAC score quantified from CCTA, it remains unclear whether the obtained errors impact clinical decision-making. Therefore, further work needs to investigate the value of the extracted CAC scores for predicting cardiovascular events.

In conclusion, an unsupervised domain adaptation method for CAC scoring that transfers knowledge from NCCT with reference labels to CCTA without reference labels has been presented. The results show that the method achieves a competitive performance. This may allow for better utilization of the existing large and annotated data sets and extend applicability to diverse CT scans without the requirement of extra annotations.

## Data availability statement

The data analyzed in this study is subject to the following licenses/restrictions: The data from NLST for this study can be requested at the provider. The cardiac NCCT and CCTA are in-home data. Requests to access these datasets should be directed to https://cdas.cancer.gov/datasets/nlst/.

## Ethics statement

The studies involving human participants were reviewed and approved by University Medical Center Utrecht; Amsterdam University Medical Center. The patients/participants provided their written informed consent to participate in this study.

## Author contributions

ZZ: conceptualized the study, developed the software, analyzed the data, and drafted the article and revised the manuscript. SV and II: conceptualized the study and drafted and revised the manuscript. NL, NP, and TL: acquired data and revised the manuscript. All authors contributed to the article and approved the submitted version.

## Funding

This work is part of the research program Deep Learning for Medical Image Analysis under project number P15-26 project 3 financed by the Dutch Technology Foundation with contribution by Philips Healthcare.

## Conflict of interest

Author II reports institutional research grants by Pie Medical Imaging, Esaote, Dutch Technology Foundation with participation of Pie Medical Imaging and Philips Healthcare (DLMedIA P15-26). Author TL reports institutional research grants by Pie Medical Imaging, Dutch Technology Foundation with participation of Pie Medical Imaging and Philips Healthcare (DLMedIA P15-26). The remaining authors declare that the research was conducted in the absence of any commercial or financial relationships that could be construed as a potential conflict of interest.

## Publisher's note

All claims expressed in this article are solely those of the authors and do not necessarily represent those of their affiliated organizations, or those of the publisher, the editors and the reviewers. Any product that may be evaluated in this article, or claim that may be made by its manufacturer, is not guaranteed or endorsed by the publisher.
