# Supplementary material for: Learning coronary artery calcium scoring in coronary CTA from non-contrast CT using unsupervised domain adaptation
Source: Front Cardiovasc Med. 2022 Sep 12;9:981901. doi: 10.3389/fcvm.2022.981901 (PMC9510682; doi:10.3389/fcvm.2022.981901)
Supplement: Supplementary file 1 [file Data_Sheet_1.PDF]

Supplementary Material

1 SUPPLEMENTARY FIGURES

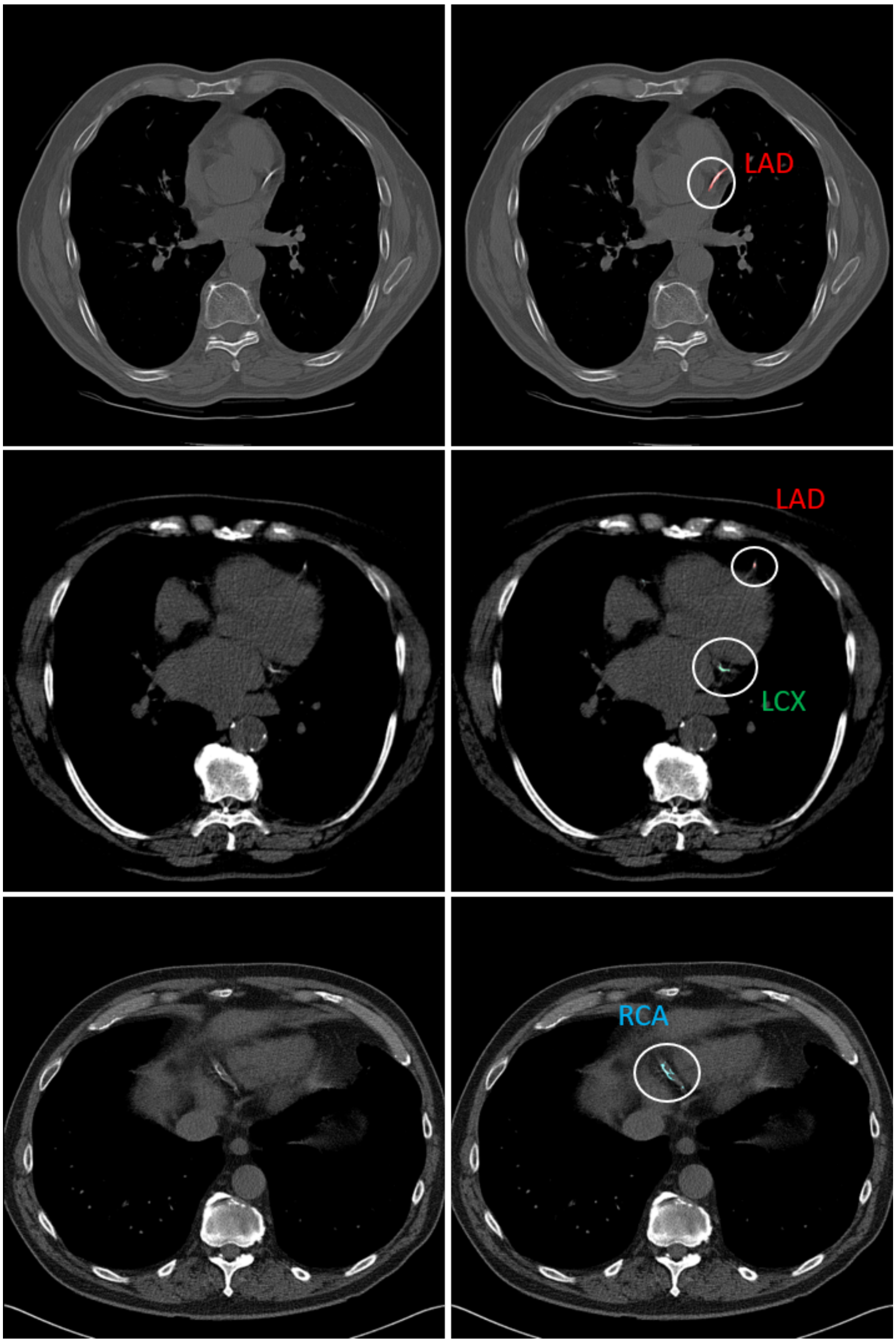

Figure S1. Three axial slices of chest NCCT scan from NLST dataset. The first column contains the CT slices and the second column contains CT slices with manual annotations of coronary artery calcium in LAD, LCX or RCA.

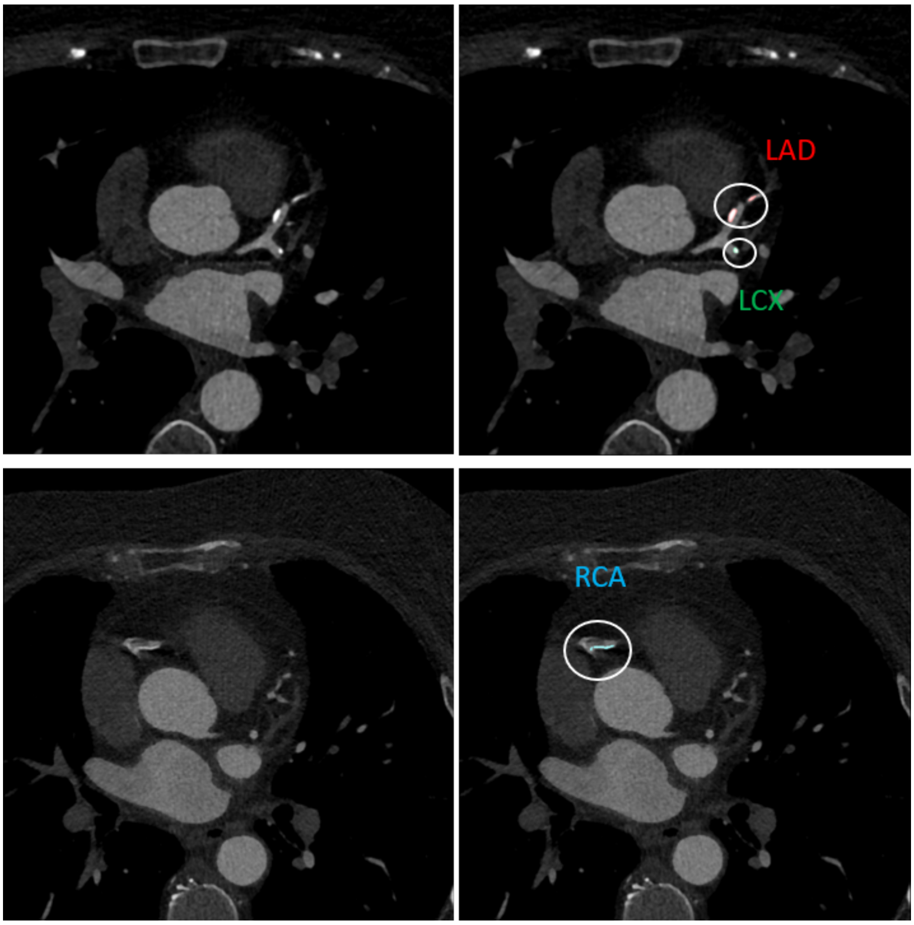

Figure S2. Two axial slices of CCTA scan from the CCTA test set. The first column contains the original CT slices and the second column contains CT slices with manual annotations of coronary artery calcium in LAD, LCX or RCA.
